# A randomized controlled trial of an Internet-based emotion regulation intervention for sexual health: study protocol

**DOI:** 10.1186/s13063-021-05586-x

**Published:** 2021-10-15

**Authors:** Vinicius Jobim Fischer, Gerhard Andersson, Joël Billieux, Claus Vögele

**Affiliations:** 1 Institute for Health and Behaviour, Department of Behavioural and Cognitive Sciences, University of Luxembourg – Campus Belval, 11, Porte des Sciences, L-4366 Esch-sur-Alzette, Luxembourg, Sweden; 2 Department of Behavioural Science and Learning, Department of Biomedical and Clinical Sciences, Linköping University, SE-581 83 Linköping, Sweden; 3 Cognitive and Affective Regulation Lab (CARLA), Institute of Psychology, University of Lausanne, uartier UNIL-Mouline – Bâtiment Géopolis – Bureau 4240, CH-1015 Lausanne, Switzerland

**Keywords:** Internet intervention, Emotion regulation, Sexual health, Randomized controlled trial, Protocol

## Abstract

**Introduction:**

Emotion regulation difficulties have been associated with mental disorders and sexual dysfunctions. Traditional face-to-face transdiagnostic emotion regulation interventions have shown positive results for emotional and personality disorders. Only recently have the effects of these interventions on sexual health started to be investigated. Internet-delivered psychological interventions have several advantages over face-to-face interventions, such as cost-effectiveness, accessibility, and suitability for people who experience shame because of their stigmatized problematic behaviors and those who avoid seeking help. The aims of the SHER 2—TREpS (Portuguese acronym for Emotion Regulation training for sexual health) project are as follows: (a) determine the efficacy of an Internet-based emotion regulation intervention for sexual health and sexual satisfaction and (b) explore the effects of the intervention on (1) emotion regulation skills, (2) mental health, and (3) sexual self-perception.

**Methods and analysis:**

The study will use a randomized controlled trial design. Eligible participants will be randomly allocated to one of two groups: intervention (Internet-based emotion regulation training) or waitlist control. Assessments will take place before the start of the trial, at the end of the trial, and at 6-month follow up, after which participants assigned to the waitlist control condition will receive the same intervention. Primary outcomes include sexual function and satisfaction and secondary outcomes self-report measures of depression, anxiety, difficulties in emotion regulation, and sexual self-perception. This intervention study is financed by the Luxembourg National Research Fund (FNS).

**Ethics and dissemination:**

Ethics approval was obtained from the Ethics Review Panel of the University of Luxembourg. Findings will be disseminated via peer-reviewed publications and conference presentations.

## Strengths and limitations of this study

- To the best of our knowledge, this is the first evaluation of an internet-based emotion regulation intervention for sexual health.
- The intervention protocol was developed encompassing both sex therapy and emotion regulation components already used for other mental disorders.
- Immediate and long-term (6 months) assessment of intervention effects on sexual health, mental health, and self-schema.

## Limitation

- Participant assessments will be conducted via self-report questionnaires.
- Self-selection of participants.

## Introduction

Epidemiological studies suggest that 40–45% of adult women and 20–30% of adult men of the general population fulfill the criteria for at least one sexual dysfunction at one point in time during their lives [1]. Sexual dysfunctions (i.e., difficulties in the ability to respond sexually or to obtain sexual pleasure [2];) are multifactorial and involve physiological, affective, interpersonal, and psychological, context-related-factors [3]. They can only be understood by considering the constituent factors of sexual health, such as the experience and expression of thoughts, fantasies, desires, beliefs, attitudes, values, behaviors, and practices [4]. These factors may play a pivotal role in the development of sexual problems, and in maintaining sexual dysfunction in the long term [5].

One important factor is emotion regulation (ER), defined as the way in which emotions are generated, experienced, and used [6]. Such processes include emotional awareness (attention, differentiation, and labeling of emotions), expression (suppression versus expression of emotions), and experience (accessing and reflecting on one’s emotions and their consequences [6]). Emotion regulation difficulties have been associated with unhealthy coping strategies, mental disorders [7], and with difficulties in the sexual response cycle of both men and women (arousal, lubrication, orgasm, pain, erection and ejaculation [8, 9]).

A range of emotion regulation interventions have been shown as efficacious in the treatment of emotional and personality disorders, e.g., the Unified Protocol (UP [10]) and the Affect Regulation Training (ART [11]). In view of the high prevalence rates of emotion regulation deficits and comorbidity of sexual dysfunctions with other mental disorders [12], such interventions might also be helpful in treating sexual dysfunctions.

Recently, two studies tested an in vivo transdiagnostic approach to sexual problems. Parsons et al. [13] conducted a pilot study with 13 HIV-positive gay and bisexual men reporting high rates of sexual compulsivity. After up to ten intervention sessions, improvements were observed in all psychological outcomes, including sexual compulsivity, depression, and anxiety, as well as decreases in drug use and HIV risk behaviors. Nonetheless, the initial small sample size (*n* = 13) and the fact that only 4 participants completed all sessions limit the conclusions that can be drawn from these results. De Ornelas Maia et al. [14] conducted an intervention to enhance quality of life and sexual functioning in unipolar depressive disorder or anxiety disorder participants. Both intervention groups (UP group intervention + pharmacological treatment, and pharmacological treatment only group) showed significant improvements in quality of life, anxiety, and depression. Improvements in sexual functioning was also noticed; the effect size was larger for sexual dysfunction in the non-depressed group (*d* = 2.62) than in the depressed group (*d* = 1.04).

Although progress has been made in the psychological treatment of sexual problems, feelings of shame in meetings with physical face-to-face contact limit their dissemination. In contrast, internet-delivered interventions may offer a greater degree of perceived privacy and, therefore, appeal to those who otherwise would avoid seeking help. Additional advantages of internet-delivered interventions concern their cost-effectiveness and accessibility [15] and the possibility for patients to deal with their problem in their home environment [16].

Internet-delivered psychological interventions vary in the way they are delivered. They are usually composed of a package of comprehensive self-help material with which the patient receives information and exercises on a weekly basis [17]. The content is delivered in the form of text, video, or audio, which is presented on a platform together with homework assignments and interactions with a clinician and/or automated support functions (i.e., self-guided treatments). Overall, there is evidence that I-therapy is more effective than no intervention and, more importantly, similar in efficacy to face-to-face treatments [18].

Internet-delivered treatments have been applied in the area of sexual health care as well. Some studies have examined the effects of addressing sexual concerns using I-therapy. In a pilot study Van Diest et al. [19] tested an Internet-delivered protocol for different sexual dysfunctions and found improvements in sexual functioning in 67% of the participants (*N* = 39), with improvements maintained at 1-month follow-up. Van Lankveld et al. [20] found that treatment was superior compared to waitlist control in an Internet-delivered sex therapy for erectile dysfunction. Similarly, Andersson et al. [21] found that 7-week internet-delivered cognitive behavior therapy significantly improved erectile performance when compared to an online discussion control group.

Recently, two studies have been registered aiming at investigating the effects of I-therapy on sexual health, one comparing two Internet-based interventions (cognitive behavior therapy and mindfulness-based therapy) for the treatment of low sexual desire in women [22] and one analyzing the feasibility of a brief online psycho-educational intervention for sexual interest/arousal disorder [23]. However, there is a lack of studies examining the efficacy of Internet-delivered emotion regulation psychological interventions for sexual health.

## Aim

The first aim of the SHER 2—TREpS study is to determine the efficacy of an emotion regulation intervention for improving sexual health and sexual satisfaction.

The second aim is to explore the effects of the intervention on factors potentially mediating its effects, i.e., (1) emotion regulation skills, (2) anxiety and depression symptomatology, and (3) sexual self-perception (sexual self-schema and automatic thoughts during sexual activity).

### Research questions

In more detail, the following research questions will be addressed:

1. Does the TREpS intervention increase sexual health and sexual satisfaction?
2. Does the TREpS intervention increase emotion regulation skills?
3. Does the TREpS intervention decrease anxiety/depression symptomatology?
4. Does the TREpS intervention improve sexual self-schema and decrease negative thoughts during sexual activity?

## Methods

### Design

A randomized controlled trial will be conducted. Participants will be allocated to either group 1 (intervention) or group 2 (waitlist).

Randomization will be performed prior to the enrolment of study participants. Two separate block randomization lists will be created (one per sex) via sealed envelope and participants with baseline completed will be allocated either on the TREPS intervention group or to a waitlist control group. The allocation will be conducted by a clinical psychologist with no involvement in the project and the intervention provider will have no influence on participants allocation. Analyses will be conducted and presented following the Consolidated Standards of Reporting Trials (CONSORT) statement [24, 25].

### Participants

#### Inclusion criteria

Individuals are eligible for participation if they meet the following criteria: (1) between 18 and 65 years of age, (2) fluent in Brazilian Portuguese, (3) self-reported sexual problems, assessed in men by a score of < 21 on the International Index Erectile Function (IIEF) and in women by a score of < 26 on the Female Sexual Function Index (FSFI), and (4) in a stable relationship for at least the preceding 3 months.

#### Exclusion criteria

Volunteers will be excluded if they report (1) medical conditions that can interfere with the outcomes of the intervention, e.g., diabetes, cancer, cardiovascular problems, or (2) ongoing psychotherapy.

### Recruitment

Recruitment will be conducted via the internet only. Advertisements concerning the project and invitations to take part in the study will be implemented using social media, targeting the Brazilian Portuguese speaking population. Additionally, participants of a previous online survey (SHER 1- study) who volunteered to participate in future studies will also be contacted.

### Participants’ information and consent

Written informed consent will be obtained electronically from all participants before any data collection ensues. The information and consent form will explain the objectives of the study, that there is no incentive for taking part, and that privacy and confidentiality is guaranteed as well as the right to withdraw from the study at any point in time without giving reasons or any negative consequences. There is no foreseeable risk associated with participation. Potential burdens from taking part in the study include the possible inconvenience from spending more time with one activity or feeling uncomfortable because of some questions or materials. In case participants would feel upset as a result of answering some of the questions or being involved in some of the intervention activities, they will have the opportunity to contact the research team, with or without disclosing their identity. In case of unexpected findings (for example concerning mental health), participants will be informed, supported, and guided by the research team.

If participants have any questions or wish to be informed of the results of the project and relevant publications, they are given with the opportunity to contact the principal investigator through the contact information provided in the online survey.

### Sample size

Considering the number of variables of the study (with a power of .80 or greater and with a significance level set at *α* = .05), the minimum sample size needed to find meaningful differences in sexual health is 102 participants (51 in the intervention and 51 the control group [26]). Considering gender differences, we will aim to obtain twice this sample size (*N* = 204), i.e., 102 participants per gender.

#### Outcomes

*Primary outcome*

1. Improvements in self-reported sexual health from baseline to 6-month follow-up.
  *Secondary outcomes*
2. Emotion regulation improvement (from baseline to 6-month follow-up).
3. Reduction in automatic thoughts during sexual activity (from baseline to 6-month follow-up).
4. Reduction in anxiety scores (from baseline to 6-month follow-up).
5. Reduction in depression scores (from baseline to 6-month follow-up).
6. Improvement in sexual self-schema (from baseline to 6-month follow-up).

### Measurements

Measurements will be undertaken at 3 time-points in each group: baseline, end of intervention, and 6-month follow up.

#### Sexual health

Sexual health will be assessed using four questionnaires, two of which concern self-perception as a sexual person, thoughts, and emotions during sexual activity and two questionnaires on sexual function.

Female participants will be asked to complete the Female Sexual Function Index (FSFI [27]) and the Sexual Quotient- female version (SQ-f [28]), while male participants will be asked to answer the International Index of Erectile Function (IIEF [29]) and the Sexual Quotient- male version (SQ-m [30]).

A) *Female Sexual Function Index (FSFI)* [27]: this is a 19-item questionnaire for the assessment of sexual functioning in women in domains of sexual functioning (e.g., sexual arousal, orgasm, satisfaction, pain). Answers are provided using a 5-point Likert scale. Hentschel et al. [31] translated and validated the he FSFI into Portuguese, showing good internal consistency both for the evaluation of the total scale (*α* = .92) and for specific domains (desire = .67; excitation = .80; lubrication = .89; orgasm = .87; satisfaction = .85; pain = .86).

B) *International Index of Erectile Function (IIEF)* [29]. The *IIEF* is 15-item, self-administered questionnaire for assessing sexual functioning in men. Answers are given on a 6-point Likert scale. The *IIEF* encompasses five different domains of sexual functioning: erectile function, orgasm function, sexual desire, intercourse satisfaction, and overall satisfaction. Ferraz and Cicconelli [32] translated and adapted the scale to Brazilian Portuguese. Its psychometric properties were assessed by Gonzáles et al. [33], showing good internal consistency for both the full scale (*α* = .89) and the specific domains (erectile function = .86; orgasmic function = .63; sexual desire = .77; sexual satisfaction = .60; general satisfaction = .73).

C) *Sexual Modes Questionnaire (SMQ)* – Automatic Thoughts subscale [34]. This self-report scale consists of 30 items in the male version and 33 items in the female version. Respondents are asked to rate the frequency (from 1 [never] to 5 [always]) with which they have experienced specific automatic thoughts during sexual activity. The psychometric properties of the Brazilian adapted version were evaluated by Lucena [35], with an internal consistency of *α* = .92 and retest reliability of *r* = .8 (*p* < .05) for the female version and *α* = .95 and *r* = .82 (*p* < .05) for internal consistency and reliability, respectively, for the male version.

D) *Sexual Quotient (QS)* [28, 30]. The *QS* is a brief and comprehensive tool composed of 10-questions, which are answered on a scale from 0 (never) to 5 (always). It addresses general sexual function and stages of the sexual response cycle (desire, arousal, orgasm) and sexual satisfaction. The female version showed excellent internal consistency, both for the questionnaire as a whole (*α* = .98) and for each of its domains (all with *α* ≥ .9) [28]. The male version showed satisfactory internal consistency for the questionnaire as a whole (*α* = .6) and for the separate domains (all with *α* ≥ .6).

E) *Sexual Self-Schema Scale (SSSS)*. Originally developed by Hill [36], the Brazilian version of the *SSSS* consists of 30 items assessing respondents’ perception of themselves as a sexual person compared to others of the same gender and age. Answers are provided using a 5-point Likert scale ranging from 1 (not at all descriptive of me) to 5 (very much descriptive of me). It has good test-retest reliability (*r* = .6, *p* < .05) and internal consistency (scale total Cronbach's alpha of .8, ranging from .61 to .85 for the three factors [35]).

#### Mental health assessment

for the assessment of mental health problems participants will complete the *Patient-Heath Questionnaire (PHQ-9)* and the *Generalized Anxiety Disorder 7* (*GAD-7)*. Both instruments are frequently used self-report diagnostic tools for the assessment of mental disorders.

a) The *Patient Health Questionnaire-9 (PHQ-9)* is a nine-item screening instrument, which also provides an assessment of the severity of depression. The diagnostic validity of the tool has been established for its English language version [37] as well as for the Brazilian version [38]. The questionnaire has good psychometric properties (77.5% sensitivity and 86.7% specificity) [39].

b) The *General Anxiety Disorder – 7 (GAD-7)* is a brief self-report measure specifically developed to assess Generalized Anxiety Disorder [40]. It has good reliability, as well as criterion, construct, factorial, and procedural validity. The Brazilian version has good internal consistency and reliability with Cronbach’s alpha of *α* = .916 and a rho composite reliability coefficient of *ρ* = .909 [41].

#### Emotion regulation assessment

For the assessment of emotion regulation, the *Difficulties in Emotion Regulation Scale (DERS)* will be used. The *DERS* is an empirically grounded assessment instrument measuring emotional regulation using a multidimensional framework, developed by Gratz and Roemer [42], and validated in Brazil by Miguel et al. [43] with its psychometric properties confirmed (*α* = .94 for the overall scale, ranging from 0.79 to 0.88 on subscales [44]). The *DERS* assesses several facets of emotion regulation, including difficulties relevant to an individual’s (a) acceptance of emotional responses, (b) ability to engage in goal-directed behavior under distress, (c) ability to control impulsive behaviors when distressed, (d) access to emotion regulation strategies, and (e) emotional clarity. Participants rate their degree of agreement with each statement on a scale from 1 (almost never; 0 to 10%) to 5 (almost always; 91 to 100%).

### Intervention

The intervention will involve an online emotion-regulation skills training for individuals with sexual problems. The protocol was developed based on existing emotion-regulation therapies [7, 10, 11, 45, 46]. It will last for 8 weeks, encompassing psycho-educational and emotion-regulation skills components. Every week participants will gain access to a different intervention module of the training, containing videos, presentation slides, written support material, and a recommendation of activities to be completed until the following week of training. Participants are expected to dedicate 30 min to 1 h per week to complete each module.

The intervention is structured as shown in Table 1.

**Table 1:** Summary of intervention modules

	Module	Content
Week 1	Psycho-education on sexual function	Covers information about sexuality, sexual response cycle (desire, excitement, plateau, orgasm, resolution), and main difficulties that men and women may face in their sexual health, such as erectile dysfunction, premature ejaculation, desire disorders, pain disorders, and anorgasmia. It also differentiates the psychological characteristics of functional sexual response from a dysfunctional sexual response as well as the difference between sexual function and sexual satisfaction.
Week 2	Psycho-education on emotions and emotion regulation	Covers the definition of what emotions are, evolutionary aspects of emotions, emotion functions, emotion response cycles, emotion components (physical sensations, thoughts and behaviors), and long-term consequences of maintaining an emotional state for a longer period of time. It also defines pleasant and unpleasant emotions, the relationship between unpleasant emotion and avoidance behaviors, and avoidance strategies (emotional suppression, distraction and behavioral avoidance).
Week 3	Relaxation strategies: breathing and muscle relaxation	Informs about the common physiological response to anxiety and stress (e.g., increases in heart rate, respiration and muscle tension) and teaches two relaxation strategies: breathing relaxation and progressive muscle relaxation.
Week 4	Cognitive flexibility	Refers to the rational component of emotion regulation. Aims to conceptualize and enhance cognitive flexibility, the triad situation-thought-emotion is explained and detailed through the concepts of what distinguishes thoughts and interpretations, what automatic thoughts and cognitive distortions are, and the identification of negative thought patterns and most common cognitive distortions related to sexuality are described.
Week 5	Non-judgmental awareness	Aims at teaching participants to experience their emotions in the present moment in a nonjudgmental way. The module also differentiates between experiencing an unpleasant emotion from experiencing an unpleasant emotion resulting from negative beliefs and reactions to experiencing it (snowball effect).
Week 6	Self-acceptance and compassion	Focuses on two psychological concepts necessary for a better emotion management: acceptance and self-compassion. These are important in order not to avoid emotional experiences and to diminish self-criticism associated with sexual difficulties.
Week 7	Emotion analysis	Presents a step-by-step flow-chart of how to identify emotions when experiencing them. By identifying emotions properly, we facilitate effective emotion regulation. The flow chart is composed of 6 items to pay attention when identifying an emotion: (1) emotion, (2) event-trigger situation, (3) evaluation/interpretation, (4) physical sensations, (5) previous similar experiences, and (6) behavior.
Week 8	Sexual emotional exposures	Makes a summary of all previous modules and suggests a series of sexual experiences paying attention to the emotions experienced during the activities (exposures). Such gradual approach diminishes the risk of intense emotions and avoidant behaviors. By succeeding on the exposures, obtaining pleasure on the activities, assessments of danger/discomfort diminish and more adaptive evaluations arise, facilitating the identification and modification of emotional behaviors.

### Control

The control group will not receive any intervention during the trial but will be offered the same treatment at the end of the 6 months follow-up assessment (waitlist-control).

### Data collection and management

All necessary precautions will be taken to maintain the confidentiality of study participants. Information collected will be pseudonymized so that individual identities cannot be revealed. For each study participant, a unique identifier will be generated and associated with his/her data records. The identifier key list containing personal identifiers (name, address, phone number, etc.) will only be accessible to the investigators of the study. The key list will be stored in locked cabinet in the premises of the researcher’s institution (University of Luxembourg) and will be destroyed after the trial has come to an end.

The servers are located in a locked computer room where only authorized personnel form the University’s IT department have access using cards and keys. Sensitive data (contact information, journals, conversations, answers to assignments) will be stored encrypted in the database, using algorithms such as AES256 with secret keys etc.; furthermore, it is not possible to establish a link between this stored data and individual users by access to the database.

All data communication between servers and users is encrypted (via TLS/https). Each participant will be assigned a random user code (four digits followed by four letters), which is used by the researcher carrying out the intervention to identify participants during the intervention. All communication related to the treatment takes place on the platform, after logging in, like in bank systems, so no confidential information is sent unencrypted by e-mail. The software on the servers is kept up to date. Backup of data is taken regularly and will be kept in a separate location from the live servers. Consent forms will contain participant’s signature and his/her unique identification number.

The controlled access to the intervention platform will guarantee the confidentiality of personal identity and the right to the protection of personal data and privacy of individuals involved in the data collection. The integrity of the study will be monitored by the Thesis Supervision Committee under which the study is being conducted.

Study results will be presented as aggregated data, with no personal information. No reference to individual participants will be made in a way that allows for identification.

### Data analysis

Data will be analyzed based on three measurements: baseline, end of intervention, and follow-up 6 months later. Analyses will be conducted to ascertain the balance on the measured covariates between the treatment and the control group.

To avoid potential post-treatment complications, such as noncompliance behavior after treatment assignment, the standard intention-to-treat estimate will be performed. To assess mean differences in the different domains (sexual health, emotion regulation, mental health, and sexual self-perception) over time and between groups, repeated-measures analysis of variance will be performed, with time (assessment point) as the within-subject factor, and intervention (group) as the between-subject factor. In case of violation of normality, changes over time in groups will be assessed with related-samples Wilcoxon tests. Multiple-imputation-based methods will be used to address missing data at follow-ups.

Intervention adherence will be monitored by the number of participants accessing the weekly training modules.

## Ethics and dissemination

### Ethics approval

The trial will be conducted according to the guidelines laid down in the Declaration of Helsinki, the guidelines of the Ethics Review Panel (ERP) of the University of Luxembourg and the European Union General Data Protection Regulation (GDPR). The study design was approved by the ERP on 26 June 2020 (ERP 20-029 SHER). In case of protocol modifications, prior approval of the University’s Ethics Review Panel will be sought and communicated to all parties concerned (trial participants and trial registries).

The study is registered on ClinicalTrials.gov (NCT04792177); all necessary information according to the WHO recommendations is provided (Table 2).

**Table 2:** WHO trial registration dataset

Data category	Information
1. Primary registry and trial identifying number	ClinicalTrials.gov NCT04792177
2. Date of registration in primary registry	29 January 2021
3. Secondary identifying numbers	–
4. Source(s) of monetary or material support	University of Luxembourg, Luxembourg National Research Fund
5. Primary sponsor	University of Luxembourg
6. Secondary sponsor(s)	FNR—Luxembourg National Research Fund
7. Contact for public queries	Vinicius Jobim Fischer, MSc, University of Luxembourg, Esch-sur-Alzette, Luxembourg; email: vinicius.fischer@uni.lu
8. Contact for scientific queries	Vinicius Jobim Fischer, MSc, University of Luxembourg, Esch-sur-Alzette, Luxembourg; email: vinicius.fischer@uni.lu Gerhard Andersson, PhD, University of Linköping, Linköping, Sweden; email: gerhard.andersson@liu.se Joël Billieux, PhD, University of Lausanne, Lausanne, Switzerland; email: joel.billieux@unil.ch Claus Vögele, Dipl. Psych., PhD, University of Luxembourg, Esch-sur-Alzette, Luxembourg; email: claus.voegele@uni.lu
9. Public title	Internet-based emotion regulation intervention for sexual health
10. Scientific title	Internet-based emotion regulation intervention for sexual health (SHER 2)
11. Health condition(s) or problem(s) studied	Sexual health, mental health, emotion regulation
12. Intervention(s)	*Emotion regulation skills training*: The intervention will consist in an online emotion-regulation skills training for individuals with sexual problems. It will last for 8 weeks, encompassing psycho-educational and emotion-regulation skills components. Every week participants will gain access to a different intervention module of the training, containing videos, presentation slides, written support material, and a recommendation of activities to be completed until the following week of training. Participants are expected to dedicate 30 min to 1 h per week to complete each module.
13. Key inclusion and exclusion criteria	*Inclusion criteria for participants:* 1. Between 18 and 65 years of age. 2. Fluent in Brazilian Portuguese. 3. Self-reported sexual problems, assessed in men by a score of < 21 on the International Index Erectile Function (IIEF) and in women by a score of < 26 on the Female Sexual Function Index (FSFI). 4. In a stable relationship for at least the preceding 3 months. *Exclusion criteria for participants:* 1. Medical conditions that can interfere with the outcomes of the intervention, e.g., diabetes, cancer, and cardiovascular problems. 2. Ongoing psychotherapy.
14. Study type	Type: interventional Study design: - Allocation: randomized. - Intervention model: parallel assignment. - Masking: none (open label) - Primary purpose: treatment
15. Date of first enrolment	10 March 2021
16. Sample size	102
17. Recruitment status	Enrolling
18. Primary outcome(s)	- Change in Female Sexual Function Index (FSFI) at 6 months (time frame: baseline, 2 months after baseline (end of intervention), and 8 months after baseline (follow-up)). - Change in International Index of Erectile Function (IIEF) at 6 months (time frame: baseline, 2 months after baseline (end of intervention), and 8 months after baseline (follow-up)). - Change in Sexual Quotient (QS) at 6 months (time frame: baseline, 2 months after baseline (end of intervention), and 8 months after baseline (follow-up)).
19. Key secondary outcome(s)	Change in Sexual Modes Questionnaire (SMQ)—Automatic Thoughts subscale (time frame: baseline, 2 months after baseline (end of intervention), and 8 months after baseline (follow-up). Change in Sexual Self-Schema Scale (SSSS) (time frame: baseline, 2 months after baseline (end of intervention), and 8 months after baseline (follow-up)). Change in Patient Health Questionnaire-9 (PHQ-9) (time frame: baseline, 2 months after baseline (end of intervention), and 8 months after baseline (follow-up)). Change in General Anxiety Disorder—7 (GAD-7) (time frame: baseline, 2 months after baseline (end of intervention), and 8 months after baseline (follow-up)). Change in the Difficulties in Emotion Regulation Scale (DERS) (time frame: baseline, 2 months after baseline (end of intervention), and 8 months after baseline (follow-up)).
20. Ethics review	Ethical approval has been obtained from the Ethics Review Panel of the University of Luxembourg (ERP 20-029 SHER).
21. Completion date	30.04.2022 (anticipated)
22. Summary results	n/a
23. IPD sharing statement	Undecided

### Dissemination plan

Study findings will be disseminated through peer-reviewed publications, conference presentations, posters, and social media channels. The research findings will provide important information concerning the efficacy of an Internet-based emotion-regulation intervention for sexual health. The outcomes of the study have the potential to establish the evidence-base for an Internet-based emotion-regulation intervention for sexual dysfunctions. The results will also contribute to the identification of the processes involved in the effects of improved emotion regulation skills on depressive and anxiety symptoms, and on sexual self-schema.

### Comment

To our knowledge, this will be the first Internet-based emotion regulation intervention to promote sexual health.

## Data Availability

After completion of the study, the results will be disseminated to the study participants and the general public via submission for publication in international peer-reviewed journals and for presentation at national and international conferences.

## Appendix 1: Participant Information Sheet and Informed Consent Form

**Subject Information sheet and Informed Consent Form**

**Title of Research Project:** “Internet-delivered emotion regulation skills training for sexual health: a randomized controlled trial”.

**Acronym of Research Project: SHER**

Dear participant,

You are invited to take part in the research project “Internet-delivered emotion regulation skills training for sexual health: a randomized controlled trial” (SHER-2).

A project developed in partnership between the University of Luxembourg (UL) and by the Linköping University (LIU), Sweden, approved by the and the Ethics Review Panel of the UL (ERP 20-029 SHER).

In this study we wish to investigate this how an online emotion regulation skills training can help to improve sexual health.

**PARTICIPATION**

By participating in this study, you will help researchers to better understand how an emotion regulation skills training is related to sexual wellbeing. The results of this study will contribute to developing better interventions to target these problems.

Before joining the study, you need to read this information form, which contains important information to assist you in deciding whether or not signing up to participate is in your best interest. If you have a question about this document that has not been sufficiently answered or explained, do not hesitate to contact the research team for more information.

To determine whether you are eligible to enter this study, you must meet these conditions: be an adult between 18 years and 65 years of age, be fluent in Brazilian Portuguese, and be in a relationship for at least the last 3 months.

After agreeing by signing the consent form, you will be asked to complete a sociodemographic form, and four questionnaires about the way how you deal with everyday emotions and your sexual wellbeing. All questionnaires are standard instruments, which are widely used in similar studies. The completion of the survey will take you approximately 10-20 minutes.

After completing the questionnaires, we will let you know if you meet the criteria for participation in the training. We will invite participants based on the score obtained in a questionnaire about sexual health. As the training aims to help people with problems, we will only invite those participants with scores indicating moderate to severe sexual health problems, while survey participants with good sexual health will not be invited. Moderate to severe problems concern the frequency and intensity of sexual difficulties, and the distress they cause. In moderate cases, sexual functions (desire, excitement, orgasm) are normally preserved while in severe cases the impairment makes it very difficult to have sexual intercourse.

If you are invited to participate in the training, you will be allocated by chance to one of two groups for the study. If not, you’ll receive a recommendation of possible helplines and psychological support centers. No one from the research team can control to which group you are assigned to. This random allocation will help us to disentangle the specific effects of the training from the effects of time or expectations anyone has when taking part in such a study.

**INTERVENTION**

The intervention consists of weekly Internet sessions over a period of 8 consecutive weeks. The sessions contain different modules of the training, encompassing an informational video, reading support material and activities to be conducted during that week. The estimated completion time per week is about 1 hour. The research team is committed to answer any questions or doubt that might arise within 24 hours.

Participants allocated to the control group will be provided with access to the intervention website after the 6-month follow-up of the intervention group. It is guaranteed that participants of both groups will have access to the same intervention.

Your participation is free, the partners of the project implement measures to protect your data detailed below, and the researchers involved in this project are bound by their professional confidentiality.

**CONFIDENTIALITY AND PROTECTION OF PERSONAL DATA**

As part of the study your personal data will be processed and analysed to achieve the scientific objectives of the study.

**What data do we collect and process?**

The data that will be collected regards your sexual health, mental health, emotion regulation skills.

All necessary procedures and precautions will be taken to maintain the confidentiality of study participants. Information collected will be pseudonymised so that individual identities cannot be revealed. For each study participant, a unique identifier will be generated and associated with his/her data collection records. The identifier key list containing personal identifiers (name, address, phone number, etc.) will only be accessible to the investigators of the study at the University of Luxembourg. The key list will be stored in locked cabinet in the premises of the principal investigator (PI’s office).

The research project, and by extension access to the intervention platform, will guarantee the confidentiality of personal identity, and the right to the protection of personal data and privacy of individuals involved in the data collection.

Study results will be presented as aggregated data, without personal information. No reference to individual participants will be made in a way that allows for identification.

**Who is responsible for the processing of my personal data?**

The Data Controller in respect of the processing of your data are the University of Luxembourg, a Public Institution of Higher Education and Research, having its registered office at 2 avenue de l’Université, L-4365 Esch-sur-Alzette, Luxembourg, acting for the Department of Behavioural and Cognitive Sciences, at the Faculty of Humanities, Education and Social Sciences and the University of Linköping.

For any request concerning the processing of your personal data you can contact the Data Protection Officer by e-mail at dpo@uni.lu, or by post at the following address:

UNIVERSITÉ DU LUXEMBOURG

Data Protection Officer

Maison du Savoir

2, Avenue de l’Université

L-4365 Esch-sur-Alzette

**How do we protect your personal data?**

You will only be identified by a code number in the data analysis and in all reports that will be produced by the research team based on this study. Exceptionally, you may be contacted by the investigators but you won’t have to disclose your name. All collected data will be used exclusively for the purposes of the study.

**How long your personal data is stored?**

Your personal data will be stored under the University of Luxembourg premises for a duration of 10 years after the publication of the results of the study.

**On what legal basis for the processing do we process the data?**

The use of your personal data is necessary to achieve the objectives of the Research project, which we are conducting in the public interest in accordance with article 6.1.e) General Data Protection Regulation 2016/679 and for scientific research in accordance with article 9.2 j).

**Who are the recipients of your personal data?**

The recipients of your personal data are the Researchers of the University of Luxembourg, involved in this research Project.

**What are your rights under the General Data Protection Regulation?**

You will have the right to access and amend your personal data. In certain cases (in accordance with the conditions set out by the General Data Protection Regulation 2016/679), you will also have the right to object to the way in which your data is used, to request that your data be deleted, to ask to restrict certain aspects of the processing of your data, and to retrieve your data to forward it to a third party (right to data portability). If you wish to exercise your rights, you should contact the principal investigator or his/her designated representative. He/she will liaise with Data Protection Officer of the University of Luxembourg. You also have the right to lodge a complaint with Luxembourg's National Commission for Data Protection (CNPD) in relation to the processing of your personal data. Further information is provided on http://www.cnpd.lu. You can also use their contact form, at: https://cnpd.public.lu/fr/support/contact.html

For exercising your rights you can contact the Data Protection Officer by e-mail at dpo@uni.lu, who will liaise to the PI of the project to handle your request. You can also proceed by post at the following address:

UNIVERSITÉ DU LUXEMBOURG

Data Protection Officer

Maison du Savoir

2, Avenue de l’Université

L-4365 Esch-sur-Alzette

**What if I do no longer want to participate in the study?**

You have the right to withdraw from taking part in this study at any time without giving reasons or any negative consequences for yourself.

**What are the risks?**

There is no foreseeable risk associated with participation. Possible burdens you may have from taking part in the study are possible inconvenience from spending more time at one activity or feeling uncomfortable because of some questions or materials. Should you feel upset as a result of answering some of the questions or doing some of the activities you have the opportunity to contact the researcher (details below), with or without disclosing your identity.

In case of unexpected findings (for example concerning mental health), participants will be informed, supported and guided by the research team.

At the end of the study, and at your request, your investigator will inform you of the overall results of this research.

If there are any questions, you can always contact the researcher, Vinicius Jobim Fischer via email: vinicius.fischer@uni.lu or by phone (+352) 46 66 44 55 78.

**ELECTRONIC CONSENT**

By clicking on the “agree to participate” box, you confirm that:

- I am aware of the content and objectives of the study and that you have voluntarily decided to take part in it.
- I have been informed that it is my right to refuse to take part in the study at any time, and that if I choose to refuse I do not have to give a reason.
- I understand that the data collected during the research would be protected in accordance to confidentiality. They can only be accessed by people subject to professional secrecy belonging to the team-investigating.

□ Agree Contact: Phone number ( )___________________

□ Disagree
